# A qualitative study on healthcare professional and patient perspectives on nurse-led virtual prostate cancer survivorship care

**DOI:** 10.1038/s43856-023-00387-6

**Published:** 2023-11-02

**Authors:** Karen Young, Ting Xiong, Kaylen J. Pfisterer, Denise Ng, Tina Jiao, Raima Lohani, Caitlin Nunn, Denise Bryant-Lukosius, Ricardo Rendon, Alejandro Berlin, Jacqueline Bender, Ian Brown, Andrew Feifer, Geoffrey Gotto, Joseph A. Cafazzo, Quynh Pham

**Affiliations:** 1 Institute of Health Policy, Management, and Evaluation, University of Toronto, Toronto, ON Canada; 2 Centre for Digital Therapeutics, University Health Network, Toronto, ON Canada; 3 Systems Design Engineering, University of Waterloo, Waterloo, ON Canada; 4 School of Nursing, McMaster University, Hamilton, ON Canada; 5 Department of Urology, Queen Elizabeth II Health Sciences Centre, Halifax, ON Canada; 6 Princess Margaret Cancer Centre, University Health Network, Toronto, ON Canada; 7 Dalla Lana School of Public Health, University of Toronto, Toronto, ON Canada; 8 Division of Urology, Niagara Health System, Saint Catharines, ON Canada; 9 Institute for Better Health, Trillium Health Partners, Mississauga, ON Canada; 10 Division of Urology, University of Calgary, Calgary, AB Canada; 11 Telfer School of Management, University of Ottawa, Ottawa, ON Canada

**Keywords:** Health services, Prostate

## Abstract

**Background:**

Virtual nurse-led care models designed with health care professionals (HCPs) and patients may support addressing unmet prostate cancer (PCa) survivor needs. Within this context, we aimed to better understand the optimal design of a service model for a proposed nurse-led PCa follow-up care platform (Ned Nurse).

**Methods:**

A qualitative descriptive study exploring follow-up and virtual care experiences to inform a nurse-led virtual clinic (Ned Nurse) with an a priori convenience sample of 10 HCPs and 10 patients. We provide a health ecosystem readiness checklist mapping facilitators onto CFIR and Proctor’s implementation outcomes.

**Results:**

We show that barriers within the current standard of care include: fragmented follow-up, patient uncertainty, and long, persisting wait times despite telemedicine modalities. Participants indicate that a nurse-led clinic should be scoped to coordinate care and support patient self-management, with digital literacy considerations.

**Conclusion:**

A nurse-led follow-up care model for PCa is seen by HCPs as acceptable, feasible, and appropriate for care delivery. Patients value its potential to provide role clarity, reinforce continuity of care, enhance mental health support, and increase access to timely and targeted care. These findings inform design, development, and implementation strategies for digital health interventions within complex settings, revealing opportunities to optimally situate these interventions to improve care.

## Plain Language Summary

Prostate cancer (PCa) survivors in Canada receive follow-up care after treatment through a specialist-led model, which is currently straining to meet patient needs. We interviewed healthcare providers (HCPs) and patients to investigate the design and development of a healthcare service that uses technology, also known as virtual care, to provide nurse-led follow-up care. Mixed experiences with virtual care informed participant feedback and concerns, including impacts of the pandemic and digital literacy considerations. We show that HCPs and patients see potential benefit in virtual nurse-led follow-up care if it can increase access to resources, clarify patient and provider care roles, and improve access and continuity of care. This type of approach to follow-up care may help to improve survivor quality of life and PCa follow-up care while extending the reach of healthcare systems with limited resources.

## Abstract (web summary)

Young et al. report qualitative findings exploring patient and healthcare provider perspectives to inform a nurse-led virtual prostate cancer survivorship clinic. While caveats regarding accessibility, clinical fit and implementation are raised, a virtual clinic designed in consideration of these factors is deemed to be acceptable and feasible.

## Introduction

In Canada, approximately one in eight males will be diagnosed with prostate cancer (PCa)^1,2^. PCa care is complex and encompasses widely varying symptoms, treatments, and side effects. For example, clinicians are tasked with the challenge of identifying clinically significant disease, without over-diagnosing or over-treating indolent disease^3^. This complexity continues in determining which treatment strategies are most likely to result in the best clinical outcomes for a patient, each with their own host of side effects^4^. With these side effects comes the risk for heightened psychological distress and reduced quality of life for survivors^5,6^. To address this complexity from within the clinical context, clinical PCa care teams are often multidisciplinary^7^, aiming to facilitate sustainable PCa follow-up care and overcome adverse long-term outcomes^8^.

In Canada, survivorship care largely focuses on monitoring for disease recurrence, as well as assessing and managing treatment-related side effects in a timely manner^9^. Patients are scheduled for visits at increasingly spaced-out intervals once they have completed curative-intent treatment^10^. While cancer survivorship commences at diagnosis, we focus specifically on the post-treatment survivorship phase for patients who have completed curative-intent treatment with no evidence of disease (Ned) or have a diagnosis but do not require immediate treatment as with patients on active surveillance or watchful waiting (i.e., monitored until their condition warrants treatment).

In traditional models of specialist-led follow-up care, three leading challenges have been described. First, care is highly reliant on a limited number of specialist physicians^11,12^. Concurrently, a growing number of Canadians are entering PCa survivorship through advances in treatment and diagnosis, resulting in high-volume clinics and considerable time burdens for these providers^13,14^. The second challenge is that monitoring for disease recurrence may not focus enough on quality of life assessment and improvement, which can be isolating and psychologically distressing^15,16^. The third challenge is that survivors often experience multifaceted physical and psychosocial side effects associated with PCa treatment (e.g., urinary function and incontinence, sexual dysfunction, bowel function, anxiety, and depression^17^). Navigating dialogue about these needs, especially those related to psychological and sexual function concerns, can be awkward and difficult for survivors and caregivers^18^. Survivors continue to report unmet needs at least 15 years post-diagnosis, which can result in wide-ranging effects to their quality of life^18^.

Recent clinical guidelines and research efforts have proposed new models of PCa follow-up care led by nurses, family practitioners, and patients, or a combination of multiple personnel beyond specialists^13^. In particular, the nurse-led model has accumulated evidence on aspects of safety^19^, effectiveness^20^, and satisfaction^21^ through both in-person and virtual modalities^22,23^. The advancement of digital health innovations (DHIs) has further provided an opportunity to improve sustainable cancer survivorship care^24^. Nurse-led DHIs have been developed for PCa survivorship self-management, symptom telemonitoring, and psychosocial care^25–28^. These solutions have shown efficacy in educational, informational and psychosocial aspects by allowing users to access more holistic support. However, their design is rarely informed by health care providers (HCPs) in practice^29^. This may be because a lack of input from HCPs during the development of DHIs exacerbates an already-challenging implementation process, which has been posited to be the result of limited clinician time and a flood of such tools entering the market^29,30^. Existing literature highlights a disconnect, as HCP perspectives are not clearly discussed in the development of DHIs^31^. HCPs may therefore face extensive barriers to the sustained use of DHIs owing to: technical limitations, limited resources to improve technological skills or digital literacy, inefficient, non-user-friendly interfaces, and poor system design^32,33^.

To ameliorate the growing mismatch between PCa follow-up care needs and the capacity of current healthcare systems, the Ned virtual clinic was developed by a consortium of patients, clinicians, and researchers at the University Health Network (UHN) in Toronto^25^. Ned was designed to optimize clinical care and patient self-management through asynchronous care delivery. It is considered a digital therapeutic, which is an “evidence-based, clinically evaluated software to treat, manage, and prevent a broad spectrum of diseases and disorders”^34^. Therefore, we refer to the Ned clinic as a digital therapeutic, the wider space as digital healthcare, and the interactions mediated by technology (e.g., person-to-person, app-to-person, person-to-app) as virtual care.

The purpose of this study follows two pillars pertaining to HCP and patient perspectives, exploring their PCa care experiences and needs in relation to the service design and development of a virtually-delivered nurse-led survivorship digital therapeutic (Ned Nurse clinic). Driven by our protocol paper^25^, our overall goal was to inform the design, development, and implementation of a virtual nurse-led model of care through research questions (RQs), posed respectively to the HCP and patient perspectives. The RQs for HCP study are, first, what barriers and facilitators to virtual care HCP perceived in their professional practice. Second, what is the role of HCPs in follow-up care delivery, the multidisciplinary nature of HCP teams, and their experiences of technology use? Finally, what are HCP perceptions of nurse-led and virtual care in the context of acceptability, feasibility, and adoption strategies? The patient study was designed to explore patients’ experiences and unmet needs are related to PCa follow-up care, patients’ experiences with and impressions of virtual PCa care, and how patients prefer their needs and preferences to be integrated into a virtually-delivered nurse-led model of PCa digital care.

Taken together, this work aims to better understand the optimal design of a service model for a proposed nurse-led PCa follow-up care platform (Ned Nurse). This study shows that the current PCa follow-up care experiences barriers in fragmented care coordination, patient uncertainty, and wait times. Participants perceive that a nurse-led clinic may be acceptable and feasible to address these barriers through coordinated care and facilitating patient self-management, when patients are supported with digital literacy considerations.

## Methods

### Conceptual frameworks

A pragmatic, human-centred design (HCD) approach was used to guide the overall study and design of the Ned Nurse (i.e., nurse-led Ned clinic) prototype^35–37^. HCD is an evidence-based, iterative process that involves and considers the needs of the end-user and perspectives of all stakeholders throughout the design process. The framework consists of three steps: (1) concept generation and ideation, (2) prototype design and system development, and (3) evaluation. This study describes phases one and two.

We adopt the stance that service design is equally as important and recursive to the technology and implementation processes that are critical to delivering and sustaining healthcare innovations into practice^38^. Here, we empathetically consider the challenges and desires that HCPs have described in their delivery of survivorship care. The Consolidated Framework for Implementation Research (CFIR, a determinant framework) and Proctor et al.’s implementation outcomes framework were applied to analyse HCPs’ and patients’ experiences with follow-up care and virtual care^39,40^. The application of these frameworks allowed us to identify possible indicators for implementation success and important considerations for the Ned Nurse service design from both perspectives. The output of this approach resulted in a readiness checklist geared to address service considerations and implementation determinants likely to lead to outcomes of implementation success for the Ned Nurse Clinic.

### Study design

This study was conducted at the UHN. Data were collected from May to June 2021. Recruitment and data collection were conducted virtually, and approval was obtained through Clinical Trials Ontario (CTO) with the UHN Research Ethics Board as the Board of Record (Project ID: 3238). This approval is a part of the larger CTO project portfolio, which maintains ethical oversight for all applicable activities associated with the Ned Nurse research programme. This study used a qualitative description design^41^ as part of a larger overall research programme for the design and development of Ned Nurse, presented according to the Consolidated Criteria for Reporting Qualitative Research (COREQ) guidelines^42^.

### Sampling

#### HCPs

To illustrate the future representative Ned Nurse HCP user, we met our goal of recruiting 10 HCPs with experience delivering PCa survivorship care via convenience sampling, set a priori according to our original study protocol^43^. We achieved thematic saturation within this sample, defined by Saunders et al. as “the degree to which new data repeat what was expressed in previous data”^44^. Clinical leads at our participating sites in Ontario (UHN, Trillium Health Partners, and Niagara Health System) reached out to colleagues from a variety of healthcare professions who delivered PCa follow-up care and gauged their interest in providing formalized feedback. Interested HCPs were connected with the study team, who assessed eligibility, collected informed consent, and interviewed participants.

#### Patients

An equal a priori convenience sample of 10 patient participants was taken, also matching our original study protocol^25^. We achieved thematic saturation within the patient sample. This sample size also yielded an equal proportion of patient perspectives compared to HCPs. Recruitment was performed through two pathways. Clinical site leaders at our participating sites in Ontario (UHN, Trillium Health Partners, and Niagara Health System) introduced the study to eligible patients. We also partnered with an external patient partner group to introduce the study and invite interested survivors to participate. Patients were deemed eligible for study participation if they self-defined as survivors of prostate cancer, were over 18 years of age, and could speak English. They were connected with our research coordinator, who introduced the study and obtained informed consent via REDCap.

### Data collection

Participant recruitment, informed consent processes, and data collection were conducted virtually with a secure video conferencing platform, Microsoft Teams (Microsoft, Redmond, Washington) due to the COVID-19 pandemic. Written informed consent to participate in the study and publish detailed quotes from interview data was obtained from all participants prior to interviews by the research team via the REDCap tool (Research Electronic Data Capture; Vanderbilt University) hosted at UHN. Data was collected via semi-structured interviews, each lasting about 60 min to meet our exploratory and feasibility goals (see Tables 1–2 for details). Interviews were audio-recorded, then transcribed verbatim via transcription software, Microsoft Word (Microsoft, Redmond, Washington). Resulting transcripts were checked for accuracy by a member of the research team. In reflection of the goals of the study, each interview consisted of several sections with corresponding questions. Please refer to Supplementary Note 1 and 2 for our HCP and patient interview guides. Patient demographic data was analysed via descriptive statistics (i.e., means and frequencies); please refer to Supplementary Table 1.

**Table 1:** HCP semi-structured interview goals and sample questions.

Goals	Example of question
Exploratory goals	
1. HCPs’ role in the delivery of PCa follow-up care	Currently, could you describe how you deliver PCa survivorship care?
2. Understanding the composition and multidisciplinarity of PCa follow-up care team	What other healthcare providers are part of your clinical team, for supporting PCa survivors?
3. HCPs’ current technology use in clinical practice	What are your experiences with digital health tools or telemonitoring systems in your clinic?
Acceptability, feasibility, and adoption goals	
1. Algorithm of the virtual nurse-led survivorship clinic (i.e., Ned)	One feature of Ned is having the system alert you when your patient may require attention. What type of information needs to be inputted into the system for such features?
2. Secondary care features of the Ned	What secondary care (i.e., psychosocial, rehabilitative, nutrition etc.) would be helpful to have indicators for?
3. Impression of nursing roles in the Ned	What direct care do you envision the nurse being able to deliver? What direct care should be triaged to the physician level?

**Table 2:** Patient semi-structured interview goals and sample questions.

Goals	Example of question
Exploratory goals	
1. Patient experiences and unmet needs with PCa follow-up care	Please describe what your experience has been like being a survivor and living with your condition?
2. Patient perceptions of their clinical circle of care (process).	What types of clinicians or healthcare providers are involved in your care (i.e., urologist, radiation oncologist, nurses, family doctor, specialists, mental health experts)?
3. Patient perceptions of a typical follow-up visit.	Is there anything you would change or improve about the follow-up visit or care you receive?
Acceptability, feasibility, and adoption goals	
1. Patients’ experiences with virtual care and impressions of a nurse-led virtual PCa care clinic (i.e., Ned)	[described Ned Nurse components of this nurse-led model of care and the flow of technology and information collected] Based on what I just told you, what are your first impressions of the Ned Nurse clinics?
2. Usability perceptions of virtual survivorship care model	What do you think about patients using this telemonitoring tool for their survivorship care?
3. Impressions of nursing roles in the Ned Clinic	What direct care do you envision the nurse being able to deliver? What direct care should be triaged to the physician level?

### Data analysis

Interview transcripts were independently analysed by three members of the research team (i.e., coders) via NVivo 12 (QSR International, Melbourne, Australia). In the first stage of analysis, deductive content analysis was performed using a codebook derived from the interview guide and elements of Proctor et al.’s implementation outcomes framework salient to the pre- and early stages of implementation^40,45^. Concurrently, new codes were also allowed to emerge inductively through discussion among the research team. Consensus on code definitions and usage was achieved through negotiated agreement. Coders began by reading each transcript to familiarize themselves with the data. Then, three transcripts were collaboratively coded in each group of transcripts to establish consensus on code definitions and usage, and the remaining transcripts coded separately. The second stage of analysis used a deductive content analysis approach to identify implementation barriers and facilitators (i.e., determinants) by participants as classified by the CFIR^39^. These determinants were mapped back to the implementation outcomes identified earlier in our analytic process to yield our readiness checklist^41,46^. Overall, we aimed to understand HCP and patient experiences with PCa follow-up care to inform service design, while identifying possible determinants (i.e. CFIR) and contextualizing their perceptions of the acceptability, appropriateness, and adoption (i.e., Proctor outcomes) of a potential nurse-led PCa virtual follow-up care system^40^.

### Positionality of the data analysis team

High-quality qualitative research is grounded in a number of elements, including sincerity^47^, which is developed through continuous self-reflexivity by researchers. The coders unfolded their positionalities to elucidate how their backgrounds, experiences, and beliefs interacted with the data and its interpretation in service of the researcher-as-instrument principle^48,49^.

Author K.Y. is a second-generation Canadian settler and cis woman of colour from a working-class background. She is a research trainee in health informatics with focus on the contexts and relationships that shape the design and implementation of health technologies. She did not complete any interviews during the data collection phase, and is only familiar with study participants to the extent that they provided personal details during their interviews.

Author D.N. acknowledged her standpoint as an upper-class, cis-gender woman of colour living in a large metropolitan area in Canada. She is a public health and health systems researcher, with a focus on patient experience and mobile health application adoption. She is a digital native with an excellent grasp of current technological trends. Prior to this study, she has not interacted or cared for any individuals living with PCa. As such, she cannot fully comprehend the experience of clinician participants who provide care to this population. She recruited and consented all study participants and observed their interviews.

Author T.J. recognizes that she is a middle-class, cis-gender woman in young adulthood. She has access to and is comfortable with technology. She received post-secondary education in the health and health informatics fields. She had previously worked in a clinical setting but had not taken care of, nor had a close connection to, anyone who lived/is living with PCa; therefore, she cannot fully grasp the challenges faced by this patient population or HCPs providing their care.

Our results are organized as follows. First, we provide context and an overview of sample demographics for both HCPs and patients. Next, we compare and contrast HCP and patient perspectives to provide a robust analysis of their experiences and perceived opportunities within PCa follow-up care, virtual care, and digital health. This was grounded in participants’ qualitative self-assessment of their usage and comfort with digital health tools within their scope of practice (for HCPs) and receipt of care (for patients). Specifically, we outline (1) challenges of PCa follow-up care; (2) opportunities for PCa follow-up care including roles, responsibilities and scoping of a nurse-led clinic, and the desire for patients to receive additional support to enhance their self-management and wellbeing; and (3) the role of virtual care through a digital therapeutic with readiness considerations.

### Reporting summary

Further information on research design is available in the Nature Portfolio Reporting Summary linked to this article.

## Results

### Participant demographics

#### HCP context and summary demographics

HCP participants came from a variety of disciplines and included eight nurses and two urology specialists. Nurse participants practiced in a variety of areas, including genitourinary, acute care, transplant, oncology, and obstetric nursing. All had varying amounts of experience (previous or current) with providing PCa follow-up care. HCP participants were recruited from publicly-funded oncology care centres at three geographic locales across Ontario; two out of three recruitment sites are tertiary academic oncology care centres that accept referrals from across the province. Further demographic and non-participation data were not collected to protect the privacy and identity of each participant, as some HCPs were the only provider of their type at their practice location at the time of data collection.

#### Patient demographics and digital device use

The average age of patient participants was 66 years old. The majority of patients self-identified as white (*n* = 7, 70%), while a smaller number identified as Black or Asian (*n* = 3, 30%). All were retired and in married or common-law relationships. All patients felt generally comfortable with their digital devices, with a preference for desktops and laptops (*n* = 6, 60%). They frequently used their digital devices for seeking information (*n* = 10, 100%), storing information (*n* = 10, 100%), communicating (*n* = 8, 80%), scheduling (*n* = 8, 80%), and leisure activities (*n* = 8, 80%). Half (*n* = 5, 50%) also used their devices for health services.

### Care coordination, patient uncertainty, and systemic resourcing gaps challenge PCa follow-up care

#### Follow-up care can be fragmented and difficult to coordinate

All HCPs spoke to their specific tasks and domains of care during their interviews, resulting in a clear pattern of follow-up tasks.

*“So as a health professional the goal is to undertake the initial consult, discuss treatment options to patient procedures and treatment to ensure that the appropriate follow up care is provided.”* (HCP008)

Immediately post-treatment, the current standard of care includes assessment of patient responses to their treatments, pain and symptom management, patient education, and psychosocial support. In the longer term, care proceeds according to specialist-designed surveillance protocols, and symptom assessment plays a major role in follow-up care. Care decisions were primarily made by assessing patient symptom severity. If these assessments were completed by non-physician HCPs, they were reported to physicians for further consultation with patients about treatment when appropriate.

*“We have… a triage line that patients can call and it can be about anything… they will speak to a nurse, report whatever is going on…then that message will get sent to the primary nurse and then the primary nurse will have to review the information. If she can answer on her own she’ll let the patient know. If she requires some sort of consultation from the physician she’ll get in contact with them.”* (HCP001)

HCPs noted that the complexity of PCa follow-up care delivery necessitates a multidisciplinary care team led by a specialist.

*“[The care team includes] physicians, nurses, physician assistants,… clinical research team and I guess administrative staff”* (HCP007)

Team members can include physicians, physician assistants, nurses, physiotherapists, dietitians, social workers, genetic counsellors, and patient care coordinators. Often, follow-up care is managed across specialties. One patient’s treatment and follow-up care may encompass surgery, radiation, and hormone therapy or chemotherapy. This complexity requires careful coordination and networking of care across these specialist teams and sometimes with external institutions, as not all hospitals are able to offer these services in one place.

*“If there is a concern… we have a day unit that patients can go to… so that’s what the nurses would have to coordinate as well as to call over and see if they can do it there. If they can’t, then we try to do it in another centre, the [redacted] centre which is also part of our cancer clinic. And if that doesn’t work then we try to do it in a peripheral clinic, like if they aren’t from [redacted], if they’re say from [redacted] we try to get it done in a clinic over there so they can get whatever care they need.”* (HCP001)

For patients receiving services from more than one place, these patients are required to travel to other hospitals to receive treatment, which can complicate inter-provider communication and patient record-keeping.

#### The current standard of follow-up care creates uncertainty for patients

Patients perceived that the current standard of follow-up care encompassed scheduled visits with their specialists (e.g., oncologists, urologists, surgeons) with bloodwork measuring prostate-specific antigen (PSA) levels, which is standard clinical protocol in Ontario^50^. These visits previously occurred in-office, but largely transitioned to telemedicine (i.e. phone calls or video-conference) during the COVID-19 pandemic. In some cases, a marked portion of active surveillance was conducted by general practitioners, who acted as the first point of care for symptom assessments. Patients felt reassured by the anchoring of the visits, as they acted as confirmation from a trusted medical professional in regards to their prostate cancer status.

“*I guess it’s just the reassurance you hear from doctors. Although I know what my PSA was, that’s fine. It’s also good to hear reassurance from the medical professional.”* (P002)

However, they also felt a lack of clarity around the current standard of follow-up care on several key expectations, which affected their perception of the quality of care they received. Some communicated that they were frustrated with unclear timelines for symptom resolution, especially if these symptoms persisted for years. Disappointment and anger were expressed when patients felt that their concerns were dismissed by their provider. They were frank about the perceived inadequacies of their care. These included different patient–provider expectations for treatments for side effects, different perceptions of symptom severity and quality of life impact, unclear timelines for the recovery of physical function, and ambiguity regarding the responsibility to coordinate care communication and health record documentation.

“*I would like to have known what was going to happen after in a lot more detail… [my specialist] said, you’re going to have some trouble with erectile dysfunction. You’re gonna have trouble with incontinence but all bodily functions will return over a period of time… but there’s no timetable given for that*.” (P006)

#### Systemic resourcing gaps persist despite new telemedicine modalities

One benefit of telemedicine spotlighted by patients was the ability to communicate with their specialist in new ways beyond in-person visits. Patients believed that enhanced communication pathways would increase their access to their specialist—particularly valuable for patients living in rural or remote settings. Additionally, asynchronous methods of communication (e.g., email) and access to blood work requisitions without an in-person visit were deemed more efficient.

*“Because of COVID, I guess everything we do right now is, initially there’s an email. You send an email and … we get a very quick response. Like within a day or so if not the same day.”* (P008)

*“When I have my PSA I’m subscribed to like I do it through Life Labs so… I don’t wait for the doc… I just go on and get it right away.”* (P010)

Some patients attested that long wait times for follow-up appointments continued to persist. Additionally, the focus on recurrence persisted through the move to telemedicine. This was compounded for those with infrequent (e.g., annual) follow-ups, who generally reported more negative experiences and perceptions about their care.

*“I would say [the telemedicine visits are] a minute, but let’s be charitable - two or three minutes. …I mean, it’s basically ‘Your numbers are your numbers. Your numbers have been good. Do you have any problems? No, I don’t have any problems right? Yeah thanks’”* (P010)

In summary, although increased access was identified as a benefit of telemedicine, patients continued to experience gaps within the current healthcare system, including a general impression that there were insufficient resources to deliver high quality care.

### Virtual follow-up care presents opportunities to support patient self-management through nurse-led care

#### Patients desire more systemic support to manage their wellbeing

Patients reported “roller-coaster” emotions during the cycle of diagnosis, treatment, and follow-up care. Anxiety and worry about cancer recurrence were most frequently mentioned. Typical coping mechanisms included mindfulness and keeping an optimistic mindset.

“*I would be really stressed out and then I would have my PSA test and then I would be even more stressed out waiting for the results and then I would get the results and then I would sort of come back down off the ledge… it was sort of through psychotherapy and through self-reflection and evaluation, I decided I didn’t want to be on that roller coaster every three to six months… I’m just gonna live today and live healthy today*.” (P004)

Patients were mindful of the “*value in taking responsibility for your health*” (P001). They reported that they strived to maintain healthy habits (e.g., exercise, diet, mindfulness, weight control, adequate sleep, social participation). This also included increasing their knowledge about living a healthy lifestyle, changing unhealthy behaviours, careful examination of medication intake, and making shared decisions with their HCPs. In particular, they described the need for connection and community through social support from family and the PCa survivor peer community.

*“Especially that we’re on things like quality of life which unfortunately, and you know, they I don’t think the industry covers that completely. Everyone talks a good game, but it’s not really covered…”* (P001)

“*Surprisingly, when you share [your diagnosis] we find out about all the other people who has had prostate cancer or knows someone who’s had prostate cancer*.” *(P004)*

Within the current specialist-led system, patients noted that symptom self-management was an informally communicated responsibility that they learned about from peer support. They felt that symptoms and after-treatment effects (e.g., erectile dysfunction, incontinence) were ignored unless they persistently sought provider attention.

*“The biggest thing I learned again through my support group was… the hospital will spend the time with you, but it’s up to you [to ask questions].”* (P002)

*“The one thing that having been involved in a, uh, with peer support groups is that you learn an awful lot about being your own best advocate.”* (P010)

#### A nurse-led clinic should focus on supporting patient self-management

Patients envisioned a nurse-led clinic as support that would provide “*somebody to… listen to your concerns and point you in the right direction*” (P005). This support included education, resources to support wellbeing, and building community through peer support. Notably, they desired additional educational support beyond what the current system provides.

*“Yeah, I think education [would] be great… You can go through and you can pull all of this stuff together on the Internet yourself. But… my clinic may have a different protocol… or slightly nuanced or something so it would be good to get it from one place.”* (P009)

*“So, if you’re in Toronto, they say there’s always the PCa support [groups] through Toronto or Oakville or whatever… Support groups you know or other information, would that be in your library too.”* (P002)

#### Scoping a nurse-led clinic should focus on the management of care and referral pathway

HCPs judged a nurse-led follow-up care model for PCa to be an acceptable, feasible, and appropriate mode of delivering care. HCPs most often indicated that a Registered Nurse (RN) would have the appropriate scope of practice to act as the central point of contact for a virtual nurse-led survivorship platform.

*“…nurses can review blood results with the patients, […] potential lifestyle modifications or ways to deal with their symptoms and in the absence of patients being unable to manage symptoms and then be able to triage to the appropriate physicians to then have a treatment put in place for those patients.”* (HCP007)

*“There are so many things that, as an example, working in a physician’s office that I’ve been able to resolve without the physician having to come in…there’s a lot that [nurses] can do, but also limited in terms of diagnosis and certain things, further assessment…even if it’s like a matter of the nurse providing the exact issues to the physician to wean down time, that’s a huge time saver.”* (HCP010)

Some indicated a preference for an Advanced Practice Nurse (APN) or Nurse Practitioner (NP).

“*I think it would be better for an RN to [make initial judgement calls through critical care], because I feel like an NP is already so busy. [The NP] could provide more diagnosis later… I would say an RN level first would be great before they move on to the next one.”* (HCP003)

However, these expressions were often accompanied by caveats regarding the amount of experience a provider might have delivering genitourinary oncology care. Above all, HCPs were clear that they viewed the scope of this role as one of assessment, management, coordination, and escalation. A nurse in this role would need to understand when to escalate care to the appropriate care provider, but would primarily manage all non-emergent situations.

*“[it’s important] those seeing lots of prostate cancer patients to have a good understanding of the type of person and things these men suffer with, or concerns. But yeah I don’t think it’s for a certain level… [Interviewer: So not necessarily tied to scope of practice but more so the oncology related experience.] I think so*.” *(*HCP007)

*“The nurse can definitely do an initial assessment… And also in terms of providing emotional support… this is something that the nurses can also be involved in. But anywhere where you would need…diagnostic measures, the testing or medications prescribed, that would something that would need to be escalated.”* (HCP002)

#### Patients want a nurse-led model to incorporate existing care connections

Patients perceived the Ned Nurse clinic as an acceptable care model and expressed their interest in the value added through this service. They hoped that this systemic change would increase the system’s ability to provide holistic care and wellbeing support.

“*What I’m hoping in this Ned is that the nurse practitioner would then take an interest in the well-being of the patient. You know, I can actually discuss what I’m going through and feel that there is going to be some follow up.”* (P001)

*“I personally would not have a problem with [nurses as the point of contact] at all. I think it’s beneficial, right? Beats every six months, I gotta say in terms of follow ups.”* (P007)

These favourable perspectives also come with important considerations about personal preferences regarding the modality of their visits and strategies to manage continuity of care. Some expressed that they would be comfortable with only seeing a nurse but would prefer one nurse for continuity of care rather than several staff rotating through the position. Others wanted to involve their specialist in their care to continue the relationship.

“*I wouldn’t mind waiting to see the doctor… I’m old fashioned that way… I guess it’s that human interaction I prefer… As time goes on [younger men will] get into more technological means of communication. They’ll get used to it, but, I guess I’m in the old fashion group. I would still prefer seeing a person*.” (P001)

### Virtual care through a digital therapeutic: assessing system and patient readiness

#### Despite an ambivalence for current digital health tools, providers see the potential

HCPs perceived potential barriers to the further spread of digital health tools within their workplaces, many as a result of their prior experiences with virtual care. The pandemic ushered in a drastic shift towards delivering many services through virtual means very quickly, most often through telehealth modalities. The cognitive load of virtual care continued to be high. HCPs found themselves needing to provide support outside their scope of clinical care, such as technology support, to patients who were not comfortable with virtual care. These factors resulted in wariness towards these tools, as trust was not established with these HCP users during the initial implementation phase at the start of the pandemic. Furthermore, HCPs did not indicate that efforts were initiated to create this sense of trust after the use of these tools became standardized. Preserving the preferred level of work and life balance was more difficult because virtual care opened up access to work from home. Others expressed anxiety that moving towards a virtual model of care would fray the therapeutic relationship between provider and patient, resulting in decreased continuity of care.

*“So using [virtual care tools]… talking and talking and talking and describing and making sure that they understand… speaking in layman terms and going over and over again, relaying results, making sure that the patient doesn’t freak out or get you know, super worried about something. It was just exhausting for me.”* (HCP010)

However, HCPs also recognized the potential for virtual care to drastically restructure care, enhance care access, and improve patient experiences. Despite ambivalence towards their current use of these tools, HCPs noted that virtual care could provide benefits to patients, such as the ability to deliver mental health support to survivors and provide them with opportunities and flexibility to seek care when needed.

*“I feel like a lot of patients feel like they don’t know what’s going on…there’s always that behind the scenes that happens with the nurses and the doctors, but the patients aren’t always in that conversation… having that ability to have continuous care [through Ned Nurse] would give them the feeling that they are in control of their care.”* (HCP003)

*“… travel can be difficult, especially for those that live somewhere more remote. So I feel it’s definitely improved access to care. It also makes it, you know, this goes along with that, but it makes it easier to follow up with a patient.”* (HCP005)

*“…the first step is that access to [an] updated evidence base information so that patients have that knowledge base and can triage themselves whatever questions they might have.”* (HCP009)

#### HCPs seek support for patient digital literacy to avoid barriers to care

Participants raised concerns regarding the feasibility and appropriateness of virtual care for patient populations. HCPs indicated that most PCa survivors are over the age of 50 and are unlikely to consider themselves digitally literate. Additionally, providers were concerned about the effect of the digital divide and the possible inaccessibility of a virtual care tool to some groups. Specific to the patient population, HCPs named concern that prostate cancer survivors are a group of older men, placing them in the digital divide due to age and gender. However, they also expressed that with proper resources and support, they were confident that patients would be able to take on the responsibility of using digital healthcare.

*“I think the patients who don’t really use that type of technology… might not be so up to doing it that way without proper enforcement and helping them learn the ropes of how to do it… but most patients are pretty receptive to doing things, in terms of reporting their symptoms because they know that it’s beneficial for their care.”* (HCP001)

#### Virtual nurse-led care facilitates self-assurance but must address accessibility

Speaking to patients’ perceptions of the appropriateness of a nurse-led model of service, they perceived the model as appropriate because: (1) they felt that nurses were a logical first point of contact, (2) the Ned Nurse clinic was compatible with their existing care workflows, and (3) they saw the value added and peace of mind of being able to connect with a nurse to manage their symptoms.

“*I think what is most important is the psychological advantage of feeling that yes, there’s someone I can come [to], I can contact and who will respond fast… It gives you, I guess a little more confidence*.” (P003)

Echoing concerns raised by HCPs, patients independently described the desire for inclusive support to mitigate care accessibility barriers. Patients indicated not only was it important to identify highly qualified personnel, but there were important equity requirements for bridging the digital literacy gap (e.g., voice recognition and guidance). Patients noted considerations needed for lower barriers for older adults and groups on the other side of the digital divide.

*“So you know, whilst it’s great, I think we just need either an engineer or somebody who is I guess quite versed in technology to be a support system for people that are not able to. [For example] somebody who has a disability with, you know, hearing or vision issues.”* (HCP010)

*“Uh, probably, literacy. like you know, virtual, computer things like that. They might not be able to do that. That’s probably the only challenge I see, but if you know the computer literate, I don’t see why it wouldn’t be a positive thing.”* (P007)

#### HCPs see the opportunity for improved continuity of care but are concerned about patient expectations and potential for burn-out

When speaking to the possible benefits of a digital health platform, HCPs identified two main considerations. First, HCPs described the requirement for potential technological capabilities to support their role, asking “will it make my job easier?” Second, they named ease of use: “is it intuitive and easy to use?” HCPs stressed the importance of ease of use for both providers and patients, with lack of usability and integration identified as a common friction point.

*“So, nurses have a lot to do on top of their plates, if it’s a daily thing and daily care that they’re already doing. If you add this to it, you’re putting [in] more work. You’d have to make it quite simple, something that’s more or less already integrated within the system of something they’re already doing.”* (HCP004)

HCPs emphasized the need for any platform to provide appropriate patient training in order to prevent provider burnout due to potential expectations around quick response times and the risk of needing to be available 24/7. Examples of boundary-setting around the limitations of the platform and service could include clarifying the hours of availability, and ensuring that patients understand that responses are manually written by a person, not instantly generated.

*“Nurses and physicians will probably have difficulty managing the time…if people are messaging at, like, night time or [when] nurses and doctors have their working hours, will they be able to separate working hours versus… their own time. [Interviewer: That’s a great consideration so sort of setting the set operational hours?] Exactly.”* (HCP003)

HCPs shared that a digital health model can support strengthening of continuity of care and communication. A follow-up platform that incorporated both scheduled check-ins and the ability for patients to contact their provider independently was identified as an optimal system. It would capture patients who might be symptomatic or are reticent to contact their provider outside of scheduled appointments.

*“I think it will also be beneficial to the patients to know that there is someone that they can easily reach out to…a relationship with that they can feel comfortable discussing these things…a go between the physicians that they may not see as regularly.”* (HCP007)

*“I see value in having scheduled video calls…. I think that that just helps situate patients [so] that they don’t fall through any cracks. They don’t get to the point where they need quick intervention if something’s caught early or if they’re proactively addressing concerns rather than having to be reactive.”* (HCP006)

## Discussion

This study explored the care experiences of HCPs in PCa care and their needs in relation to the service design and development of a virtually delivered nurse-led survivorship digital therapeutic, as well as barriers and facilitators to virtual care in their professional practice.

HCP participants experience considerable barriers to adopting DHIs, especially in the context of the rapid pandemic-related introduction of most virtual care. We suspect that this is because of their lack of interaction and input within the design of DHIs, which is consistently a main barrier to implementation^33,51^. In true human-centred design form, users should instead be involved in all stages of design, from conceptualization to feasibility testing and implementation^52^. Involving HCPs throughout the early phases of mapping complex processes within current follow-up care models and soliciting feedback informs how to optimally situate a nurse-led clinic in this ecosystem. This is in line with existing findings across studies in other fields^7,53,54^ where HCPs have a broad scope of responsibilities^54^.

Tailored care is fostered through multidisciplinary teams and shared decision-making between HCPs, patients, and their family. It also facilitates greater patient adherence to care plans and higher-quality care^7^. It is clear that more supportive efforts are needed to galvanize the adoption and sustainability of DHIs within HCP scopes of practice. The immediate and rapid dissemination of telehealth into healthcare during the pandemic has left HCPs with a sense of ill preparation for the use of DHIs. Moreover, it is evident that HCP guidance and endorsements are key to digital health adoption and sustainability within patient populations. Overlooking the involvement of HCPs in these processes has far-reaching consequences for the digital health ecosystem as a whole.

From the patient perspective, barriers described within the current model of specialist-based follow-up care are consistent with literature regarding limited resources, long wait times, fragmented care, ability to respond in a timely manner, and the need for improved supportive care resources^15,16^. Further, we provide patient recommendations on important aspects to consider in the design, development, and implementation of patient-facing digital health tools. Benefits of telemedicine include larger geographic reach, asynchronous communication with providers, and flexibility. This flexibility can also include allowing patients to submit patient-reported outcomes (PROs) when needed, at their own pace, without feeling rushed during irregular appointments.

However, the digital divide shows that digital accessibility and its benefits accumulate for younger, higher socioeconomic-status, and white individuals^55^. Incidence of PCa increases with advancing age, which is correlated with lower digital literacy^56^. However, the self-confidence to make health decisions based on online information appears to be the largest factor in health-related internet use for PCa survivors^57^. We acknowledge that such an intervention cannot address all issues, but involving patients in the design of the care tool and service change, and providing accessible technical support to patients appears key to ensuring that any patient-facing digital tool proceeds beyond the pilot stage of implementation.

In terms of facilitators, HCPs describe five for sustainable use of DHIs: (1) ease of use, (2) patient expectation-setting and coaching, (3) proper training and provision of support to HCPs involved in providing services through the technology (4) adequate staffing, resourcing, and patient support, and (5) building trust in the platform for sustainable application of a digital health platform. These results have been echoed in studies examining HCP involvement in the digital delivery of mental health therapies^58,59^, advocacy for patient use of digital health tools^29,60^, nurse-led telehealth prostate cancer supportive care^54^, digitally-mediated therapeutic adherence efforts^61,62^, and improving patient access and adherence to digital therapeutics^63,64^. More specifically, HCPs propose a specific scope for a nurse-led DHI for PCa follow-up care and acknowledge that this nurse role is noteworthy, considering the specialist focus in current models. HCPs indicate a nurse-led model is acceptable, appropriate, and feasible if positioned as a comprehensive platform to provide patient assessment, coordination, and management.

Similarly, in considering the value-add of a virtual nurse-led model of care, patients anticipate the following factors will facilitate use: (1) clarity of roles and responsibilities; (2) ease and accessibility of use; (3) enhanced mental health support, and (4) reinforcement and continuity of care. The promise of virtual care for patients is in its ability to provide more timely and targeted care. With a lack of capacity in the current specialist-led model, a nurse-led model may be better positioned to resolve challenges in providing this care.

Existing care models for PCa and cancer survivorship primarily pursue the goal of improving clinical survivorship outcomes (e.g., survival), not patient-reported outcomes (e.g., quality of life)^65^ We suggest that a digital health survivorship platform should effectively integrate perspectives from all affected stakeholders for better communication and resource management including HCPs, patients, policy makers, and designers. From the facilitators listed above, we synthesized six HCP and five patient aspects to support sustainable uptake of virtual care moving forward guided by participant perspectives and Proctor’s implementation outcomes^40^. These aspects, expanded on in readiness checklists presented in Tables 3–5, mapped onto the CFIR and Proctor outcomes^40^ as follows: (1) functionality testing (acceptability), (2) technical support, and usability (adoption), (3) fit-for-purpose (appropriateness), (4) resource allocation (cost), (5) staff and patient readiness (feasibility), and (6) staff and patient training (penetration). These readiness checklists can be used to assess the health ecosystem to understand where resources and support are needed for the early stage of digital therapeutic development and implementation. While grounded in our results within the PCa context, these readiness checklists can be used broadly to assess the health ecosystem from the HCP and patient perspectives. They are not meant to be an exhaustive tool, but can act as a starting point to identify strengths and areas requiring additional support to improve acceptability, appropriateness, and feasibility of a new digital therapeutic. Ensuring ecosystem readiness for new digital therapeutics can improve the odds of implementation acceptability, appropriateness, and feasibility.

**Table 3:** HCP and patient implementation readiness checklist: acceptability and adoption.

Determinants	Item	Description and designing for outcomes
Functionality Testing Proctor Outcome: Acceptability	HCP • Has the platform been user-tested for technical and clinical fit and function?	As a result of the broad and rushed transition to telemedicine during the COVID-19 pandemic, HCPs are wary of new digital therapeutics. However, they also recognize the potential for digital health tools to improve care—if designed and implemented properly. Functionality testing assessment could include methods such as think aloud usability testing and heuristic evaluations by technical and clinical experts (e.g., refs. ^68,69^). CFIR Construct (Domain): Innovation Deliverers (Individuals), Assessing Needs (Implementation Process), Assessing Context (Implementation Process)^70–72^
	Patient • Has the platform been user-tested for accessibility?	Patient participants are concerned that lower levels of digital literacy within the community would affect usage of a digital therapeutic for PCa follow-up care. Accessibility could be enhanced through digital literacy evaluations, accessibility evaluations, or site-specific pilot implementations (e.g., ref. ^73^). Usability can be assessed by usability testing with patients via think-aloud and other user testing methods (e.g., ref. ^68^). CFIR Construct (Domain): Innovation Recipients (Individuals), Assessing Needs (Implementation Process)^72,74^
Technical Support and Usability Proctor Outcome: Adoption	HCP • Are patients and HCPs able to access technical support and information to guide their use of the platform?	HCPs were frustrated by a lack of technical and implementation support within their telemedicine experiences. Technical support needed can be assessed by usability testing with patients and HCPs via think-aloud and other user testing methods (e.g., ref. ^68^)
	• Has the platform been user-tested for ease of use?	HCPs felt that telemedicine modalities made their jobs more difficult during the pandemic, as a lack of technical support impacted their ability to care for patients. Usability assessment could include digital literacy evaluations, accessibility evaluations, or site-specific pilot implementations (e.g., ref. ^73^). CFIR Construct (Domain): Structural Characteristics (Inner Setting), Assessing Context (Implementation Process)^71,75^
	Patient • Does the system accommodate various stages of survivorship or disease progression? • Are resources tailored for different stages? • Has the platform been user-tested for usability?	Patients have varying survivorship self-management needs that require different responses. Ability to tailor care could be assessed by cataloguing the resources that are available to patients and categorizing these resources based on ability to deliver and what needs they are meant to serve. CFIR Construct (Domain): Innovation Recipients (Individuals), Assessing Context (Implementation Process), Innovation Adaptability (Innovation)^71,74,76^

**Table 4:** HCP and patient implementation readiness checklist: appropriateness and cost.

Determinant	Item	Description and designing for outcomes
Fit for Purpose Proctor Outcome: Appropriateness	HCP • Have appropriate use of the system and system boundaries been discussed with patients?	HCPs noted that the use of telemedicine and telework resulted in less work-life balance. Implementing direct contact with providers requires expectation management of: appropriate frequency of communication, and defining “timeliness” of expected response. This would be clinic/clinician specific. Some clinicians may support shorter or longer times for follow-up. Boundaries should be communicated to patients. CFIR Construct (Domain): Culture (Inner Setting), Innovation Deliverers (Individuals), Innovation Adaptability (Innovation)^70,76,77^
	Patient • How has the system been assessed for appropriateness to meet specific population needs?	Patients envisioned a nurse-led clinic as valuable support for self-management. Fit for purpose could be assessed by implementing the system through a pilot project and completing an assessment via suitable methodologies, based on what outcomes the system is intended to improve or support. CFIR Construct (Domain): Innovation Recipients (Individuals), Innovation Adaptability (Innovation), Innovation Trialability (Innovation)^74,76,78^
Resource Allocation Proctor Outcome: Cost	HCP • Have specific resources and staffing been allocated to the platform to ensure that it is not resulting in an increased workload for providers?	HCPs were concerned that the introduction of a digital therapeutic would add more responsibilities into their role (“pain”) without much value (“gain”). Resource allocation assessment could include qualitative assessment of whether HCPs feel they have the capacity and resources to use this platform. CFIR Construct (Domain): Available Resources (Inner Setting)^79^

**Table 5:** HCP and patient implementation readiness checklist: feasibility and penetration.

Determinant	Item	Description and designing for outcomes
Readiness Proctor Outcome: Feasibility	HCP Readiness • Are staff ready to participate in implementing this change in practice?	HCPs were not prepared for the pandemic transition to telemedicine. Staff readiness assessment could include qualitative assessment of whether they feel empowered to participate in this practice change, and that their institutional culture supports this change. CFIR Construct (Domain): Culture (Inner Setting), Tension for Change (Inner Setting), Planning (Implementation Process)^80–82^
	Patient Readiness^a^ • Do patients feel prepared for onboarding?	Patients are confused about expectations for self-management and would like clarity regarding what role they need to play in their care. Patient readiness could include qualitative assessment of whether they feel empowered to participate in this change in their care, and if they feel that their care provider supports this change. CFIR Construct (Domain): Innovation Recipients (Individuals), Planning (Implementation Process)^74,82^
Training Proctor Outcome: Penetration	HCP Training • Have staff been trained on this platform, and will they be able to access enhanced post-implementation support when using it?	Staff training assessment could include qualitative assessment of whether they feel adequately prepared to independently use the platform for its stated purpose (e.g., refs. ^83,84^). CFIR Construct (Domain): Available Resources (Inner Setting), Access to Knowledge & Information (Inner Setting)^83,84^
	Patient Training • Have patients received sufficient and appropriate training to onboard and use this platform?	Patient training could be assessed by investigating barriers and facilitators to technology adoption by patients and designing mitigating strategies for barriers. (e.g., refs. ^85–88^). CFIR Construct (Domain): Assessing Needs (Implementation Process), Tailoring Strategies (Implementation Process)^72,89^

^a^Patient readiness should be further specified based on patient needs assessment.

We review some strengths and limitations of our work as follows. Our qualitative description design allowed for the deep exploration of HCP and patient experiences and needs within PCa follow-up care, nurse-led care, and virtual care models. This allowed us to synthesize a readiness checklist that spotlights facilitators identified as important to HCPs and patients. Lessons from these provider- and patient-focused themes, with resulting pragmatic service design recommendations, can be transferred to develop and implement future evidence-based, service-oriented digital therapeutics for PCa survivorship.

The focus of the HCP perspective of this study provides the missing piece complementing patient-centred virtual care research facilitating holistic HCD for technology-based care^52^. These implications should be interpreted with the following considerations. The limited availability of practitioners and ethical concerns regarding the identification of some HCPs restricted our ability to provide demographic information. Additionally, interviews were conducted in English at academic healthcare centres that are located in multicultural urban areas situated in an industrialized country. The HCPs interviewed in this sample are highly trained and work in a high-income country setting. Limited services and the potential inequitable allocation of healthcare resources across less-resourced communities, settings, and countries are beyond the scope of our discussion^66^.

Similarly, our patient findings and implications are based on data collected in a developed, high-income setting. Transferability for developing and/or low- or medium-income settings may be more limited because of context characteristics. Additionally, prostate cancer survivorship is impacted by demographic factors such as race and ethnicity^67^; these factors were not explicitly investigated within this study. This study was conducted during the beginning of the COVID-19 pandemic when virtual care was first widely introduced in the context of a public health emergency. Our data collection phase aligned with the implementation of virtual care modalities across broader health contexts, a systemic change that influenced participant experiences. However, the immediacy of these experiences may have increased patients’ motivation to participate in designing an accessible digital therapeutic.

This study will inform an integrated care system that addresses both patients’ and HCPs’ needs via a digital, nurse-led PCa follow-up care platform. Future studies should examine findings from other settings to validate the recommendations for DHIs and facilitate the evolution of integrated and structurally inclusive systems. This will benefit patients across communities and demographic settings globally. Second, perspectives from all stakeholders should be investigated to extend a holistic view of building human-oriented, highly adopted and well-accepted digital therapeutics. To garner additional insight on readiness and feasibility, additional work would strengthen implementation efforts from the following perspectives: administrators and clinical managers overseeing environmental readiness as well as NPs and APNs who execute on implementation. Research on the early stages of service design should investigate strategies to meet comprehensive care goals across perspectives while maintaining accessible design features. DHIs should be informed by understanding the experiences of key stakeholders and recommendations from this and other exploratory investigations. Moreover, future investigation of DHIs should address important considerations beyond feasibility, appropriateness, acceptability, and accessibility. More efforts are needed to build DHIs with efficacy, sustainability and scalability for a stronger healthcare system.

## Conclusion

We investigate the experiences of HCPs and patients with PCa follow-up care to inform the design and implementation of digital PCa survivorship management models in care systems. DHIs continue to experience challenges related to adoption and acceptance. Our findings indicate that a nurse-led model of PCa follow-up care is generally acceptable to HCPs if it allows for patient assessment, management, and coordination of care by nurses. Although HCPs express ambivalence regarding the use of DHIs within their current environments, they recognize the potential for a digital therapeutic leveraging technology to increase access to mental health supports, community support, and patient resources. This study also reveals that virtual care modalities currently used in PCa follow-up care continue to underdeliver on patient care expectations. Patients want virtual care to incorporate existing care connections, support self-management, and address accessibility. This study illuminates the potential of employing technology in PCa survivorship care to expand access and coordinate resources to better support PCa survivors. We develop a health ecosystem readiness checklist for virtual PCa care platforms that considers implementation determinants from both perspectives. Further validation of this checklist is warranted to show its transferability, as well as additional work to evaluate the efficacy of digitally mediated nurse-led PCa survivorship care models and explore specific human-informed design considerations for better user uptake.

### Supplementary information

Supplementary Information

Reporting Summary

## Data Availability

Broader access to the data reported in this analysis is restricted; data sharing was not part of the informed consent agreement. Data sharing may be permitted provided additional ethical approval is sought and approved, and participants are re-consented to secondary use of their data. To request access please contact the corresponding author Q.P. (q.pham@uhn.ca).
