# Supplementary material for: A qualitative study on healthcare professional and patient perspectives on nurse-led virtual prostate cancer survivorship care
Source: Commun Med (Lond). 2023 Nov 2;3:159. doi: 10.1038/s43856-023-00387-6 (PMC10622495; doi:10.1038/s43856-023-00387-6)
Supplement: Supplementary file 1 — Supplementary Information [file 43856_2023_387_MOESM1_ESM.pdf]

Supplementary Information:

A qualitative study on healthcare professional and patient perspectives on nurse-led virtual prostate cancer survivorship care

Karen Young[1,2], Ting Xiong[1,2], Kaylen J. Pfisterer[2,3], Denise Ng[2], Tina Jiao[2], Raima Lohani[2], Caitlin Nunn[2], Denise Bryant-Lukosius[4], Ricardo Rendon[5], Alejandro Berlin[6], Jacqueline Bender[1,6,7], Ian Brown[8], Andrew Feifer[9], Geoffrey Gotto[10], Joseph Cafazzo[1,2], Quynh Pham[1,2,11]*

[1] Institute of Health Policy, Management, and Evaluation, University of Toronto, Toronto, Ontario, Canada
[2] Centre for Digital Therapeutics, University Health Network, Toronto, Ontario, Canada
[3] Systems Design Engineering, University of Waterloo, Waterloo, Ontario, Canada
[4] School of Nursing, McMaster University, Hamilton, Ontario, Canada
[5] Queen Elizabeth II Health Sciences Centre, Department of Urology, Halifax, Nova Scotia, Canada
[6] Princess Margaret Cancer Centre, University Health Network, Toronto, Ontario, Canada
[7] Dalla Lana School of Public Health, University of Toronto, Toronto, Ontario, Canada
[8] Niagara Health System, Thorold, Ontario, Canada
[9] Trillium Health Partners. Mississauga, Ontario, Canada
[10] University of Calgary, Calgary, Alberta, Canada
[11] Telfer School of Management, University of Ottawa, Ottawa, Ontario, Canada

*Corresponding author:
Quynh Pham
q.pham@uhn.ca
Centre for Digital Therapeutics, Toronto General Hospital, R. Fraser Elliott Building, 4th Floor, 190 Elizabeth Street, Toronto, Ontario, Canada, M5G 2C4

This file includes:

Supplementary Note 1. Healthcare Provider Interview Guide
Supplementary Note 2. Patient Interview Guide
Supplementary Table 1. Demographic Characteristic of Patient Participants

Supplementary Note 1. Healthcare Provider Interview Guide

Part I - Background on the Participant

General Role in Care Delivery

- *Goal - Understanding the healthcare providers' current role in survivorship care delivery*
  - *What is your position? How long have you been doing this job?*

- *Please describe the type of clinic you're working in?*
  - *What is the average clinic size?*
  - *How often does the clinic run?*
  - *What type of providers are part of your clinical team?*
- *Currently, could you describe how you deliver PCa survivorship care?*
- *On average, how many PCa Survivors do you see in a day? How long do these follow-up visits take?*

<u>*Interdisciplinary Team*</u>

- *Goal - Understand the clinic and other care professionals the participant works with*
  - *What other healthcare providers are part of your clinical team, for supporting PCa survivors? (i.e. Nurses? GPs? Mental Health Experts?)*
  - *In your clinic, what roles and responsibilities do you take on directly, and what get triaged to others? How is this decision made?*

<u>*Understanding their current technology use*</u>

- *Goal - Understanding the participants current familiarity with digital health tools*
  - *Are you currently using any digital health tools, or Telemonitoring systems, in your clinic?*
  - *If yes, what have your experiences been like with these tools? What have the challenges and barriers been? The facilitators?*
  - *If no, is there a reason as to why you have not implemented them? Any concerns? Are you open to incorporating virtual care into your practice?*

<u>Part II - Virtual Care and a Nurse-led Survivorship Clinic</u>

<u>*The Algorithm*</u>

The Virtual Nurse-led Survivorship Clinic centers on the development of a robust algorithm that can, in real-time, process incoming information and stratify patients based on issues such as risk, need for care, type of care required, etc.

- *The goal of the virtual clinic is to be able to provide the right care to the right person, at the right time. Part of this is having the system alert you when your patient may require attention. In your opinion, if you were using a system that was remotely monitoring PCa Survivors, what changes would you like to be alerted to?*
  - *i.e. If their PSA levels are increasing? If they require secondary support (i.e. psychosocial)?*
- *For the above alerts, how do you think a patient should be reporting issues before the system alerts you? I.e. 1-day, 1-week?*
- *In order to produce these alerts what type of information needs to be inputted into the system?*
- *For this information that is reported by the patient, how frequently should they be*

providing information (i.e. completing ePROs) into the system?

- *What different categories of alerts should the system include (i.e. critical vs. non-critical)? What should the definitions be for each category?*
- *How would you like to communicate with the patient if there is an alert (through the app? Phone call? Email?)*
- *Should the patient be informed of the alerts they are triggering? If so, what type of alerts should they be made aware of?*

Secondary Care

- *What secondary care (i.e. psychosocial, rehabilitative, nutrition etc.) would be helpful to have indicators for?*
- *What are the common secondary care pathways for prostate cancer survivors you have come across with your patients?*
- *Would it help if an algorithm suggests optimal follow-up pathways?*

Nursing Role

- *What direct care do you envision the nurse being able to deliver? What direct care should be triaged to the physician level?*
- *In your clinic, what is the current role of nurses (if any) in prostate cancer survivorship?*
- *What type of nurse (RN, oncology nurse, etc) do you believe would be able to best deliver this care?*

Supplementary Note 2. Patient Interview Guide

Thank you for taking the time to speak to us about your experience as someone with lived experience related to prostate cancer. We are researchers from the University Health Network and the objective of this study is to develop a nurse-led virtual clinic that will support prostate cancer patients in the survivorship phase of their cancer trajectory. As part of this research, we are conducting one round of interviews with people and caregivers of people living with prostate cancer.

The questions for this interview are divided into 2 sections:

1. *I will ask you questions about your experience living with prostate cancer.*
2. *I will ask questions to get your opinion about the virtual clinic we are developing.*

The purpose of the questions is simply to guide the conversation. However, because the goal is to understand your experiences, you should feel free to talk about anything you think might be important. You can also ask me to repeat or rephrase a question if you don't understand. Finally, you also have the choice to ask to skip a question or end the interview at any time.

The interview should last about one hour and will be audio recorded to help with our analysis. Only members of the study team will have access to the transcripts of this conversation; we will not be sharing it with your healthcare providers. Do you have any questions?

<u>Section 1 – Patient experience living with and managing prostate cancer</u>

In this first section, I'm going to ask you questions about your survivorship experience and living with and managing any condition or treatment-related symptoms, and the types of supports you get from your healthcare providers and the healthcare system.

- Please describe what your experience has been like being a survivor and living with your condition?
  - How long have you been living with your condition?
  - What kind of follow-up care are you receiving today?
  - How often do you receive follow-up care?
  - How are these visits occurring (i.e. in-person, phone visits, video visits)?
- What types of clinicians or healthcare providers are involved in your care (i.e., urologist, radiation oncologist, nurses, family doctor, specialists, mental health experts)?
  - Do you receive any support from allied healthcare providers (i.e. psychosocial support, rehabilitation support, home care, personal support worker, etc.)
  - How often do you see each of these different healthcare providers?
  - Do you know if there is any communication between your different healthcare providers? For example, if one of the doctors changes your medication, will the others know about it?
- Who is the first person you contact if you have questions about your health or if you think your symptoms are getting worse?

    - How do you usually communicate with them (e.g. phone, in person visits, email, video chat)?
    - What do you do if you can't get in contact with them?
- Tell me about what you do on a regular basis to help maintain your condition and/or general good health (exercise, diet, take medications, look up information about your condition, go to regular appointments, etc.)
    - Do you feel confident that you know the things that you should be doing to manage your condition/health? Why/why not?
    - Do you feel that you have all the information you need to take care of yourself? If not, what type of resources or information do you feel is missing in your care?
- Do you receive any support from a family member or friend in the management of your condition? In what ways do they support you?

Please describe a typical follow-up visit from your perspective

- What aspects of the visit are most valuable to you?
- Is there anything you would change or improve about the follow-up visit or care you receive?
- Overall, how has your follow-up experience been for you?

<u>Section 2 – Developing a virtual nurse-led survivorship clinic</u>

As I mentioned earlier, we are speaking with patients who will help inform the design of a virtual nurse-led survivorship clinic that will better support the clinical management and survivorship experience of prostate cancer survivors. These will be called Ned Nurse clinics (Ned = No evidence of disease). Ned Nurse clinics will have two key components:

- *The first is that there will be a trained nurse (or nurse practitioner) who will provide holistic survivorship care that is tailored to patient needs and will act as a point of contact for the patient to address prostate-cancer or QoL-related health needs. This means that this nurse will provide survivorship care for patients in-between their scheduled specialist follow-up visits, and will triage care as clinically necessary.*
- *The second component is the Ned telemonitoring technology that will support the patient in their survivorship care. So through the Ned patient application, which is web-based, patients will complete a monthly health survey that records symptoms related to their prostate cancer – this survey is called the EPIC-CP, which measures urinary function, bowel function, sexual function, and hormonal function. Survey results will go to the nurse, who will review symptoms the patient is experiencing, determine next steps in the patient's care plan and send a Nurse's Note through the application to the patient. This Nurse's Note could include either symptom self-management resources, or if the nurse thinks an appointment is necessary, the note will indicate this as well, again depending on the symptoms the patient is experiencing.*
- *Alongside this EPIC-CP survey, patients will be also able to complete another survey that is intended to be more holistic and capture QoL-related issues, such as practical needs, emotional needs, lifestyle needs etc. This survey is called the Ned Needs Assessment, and patients will be able to complete this every month along with the wellness survey.*

Completion of this survey is optional, and it will also alert the nurse if the patient is experiencing issues that they would like to speak to the nurse about.

Overall, the goal of the Nurse Ned clinic is to provide the right care to the right person, at the right time. This will support the ability for patients to be more holistically supported in their survivorship phase as well as optimize clinician management of survivorship care delivery.

Based on what I just told you, what are your first impressions of the Ned Nurse clinics?

- What aspects of survivorship care management could be enhanced with virtual care? (i.e. improved access to timely care? health education? help with self-management, better communication with care providers, etc.)?
    - Is there anything you don't like about the idea?
- What challenges do you think patients will face in using this telemonitoring system?

What do you think about patients using this telemonitoring tool for their survivorship care?

- Do you have personal experience with these types of tools?
    - If so, what has been your experience?
- Do you feel that you would have the time and/or ability to take health measures needed for your follow-up care (i.e. completing the survey every month) when prompted to do so through the telemonitoring technology? Why or why not?
    - What health measures would you want to be included in the holistic survey (i.e. Ned Needs Assessment) that you would want the Ned Nurse to know you are experiencing? How often would you want to take these measures for your condition(s)?

What do you think about the idea of having a nurse be the point of contact for reviewing telemonitoring data and communicating with the different members of your care team?

- Which healthcare providers would it be most important for the nurse to stay in contact with (e.g., the regular specialists, your family doctor, home care, etc.)?
    - Would you want this nurse to have scheduled check ins with you (i.e. every 2 months) or would you rather only be in contact with them if you're concerned about your health? If so, how often?
- If you felt that your symptoms were getting worse or that you had a question about your health, do you think you would call this Ned Clinic nurse or would you call the person you normally would before?

Supplementary Table 1. Demographic Characteristic of Patient Participants

Demographic variable	Value
Age, M (SD)	66 (5.94)
Race/ethnicity	
White	7 (70%)
Black/Asian/Other	3 (30%)
Residential setting	
Urban	5 (50%)
Suburban/rural	5 (50%)
Marital status, married/common-law relationship	10 (100%)
Employment status, retired	9 (90%)
Preferred devices	
Desktop/laptop	6 (60%)
Smartphone	2 (20%)
Tablet	2 (20%)
Comfort with preferred device	
Very comfortable	6 (60%)
Comfortable	4 (40%)
Frequency of preferred device use	
Frequently	8 (80%)
Sometimes	2 (20%)
Activities with preferred device	
Communication	8 (80%)
Information	10 (100%)
Scheduling	8 (80%)
Information storage	10 (100%)
Leisure	8 (80%)
Health	5 (50%)
