# Supplementary material for: A qualitative study on healthcare professional and patient perspectives on nurse-led virtual prostate cancer survivorship care
Source: Commun Med (Lond). 2023 Nov 2;3:159. doi: 10.1038/s43856-023-00387-6 (PMC10622495; doi:10.1038/s43856-023-00387-6)
Supplement: Supplementary file 2 — Reporting Summary [file 43856_2023_387_MOESM2_ESM.pdf]

nature portfolio

Corresponding author(s): Quynh Pham

Last updated by author(s): Oct 4, 2023

Reporting Summary

Nature Portfolio wishes to improve the reproducibility of the work that we publish. This form provides structure for consistency and transparency in reporting. For further information on Nature Portfolio policies, see our Editorial Policies and the Editorial Policy Checklist.

Statistics

For all statistical analyses, confirm that the following items are present in the figure legend, table legend, main text, or Methods section.

n/a	Confirmed	
☐	☒	The exact sample size (*n*) for each experimental group/condition, given as a discrete number and unit of measurement
☒	☐	A statement on whether measurements were taken from distinct samples or whether the same sample was measured repeatedly
☐	☒	The statistical test(s) used AND whether they are one- or two-sided<br>*Only common tests should be described solely by name; describe more complex techniques in the Methods section.*
☒	☐	A description of all covariates tested
☒	☐	A description of any assumptions or corrections, such as tests of normality and adjustment for multiple comparisons
☒	☐	A full description of the statistical parameters including central tendency (e.g. means) or other basic estimates (e.g. regression coefficient) AND variation (e.g. standard deviation) or associated estimates of uncertainty (e.g. confidence intervals)
☒	☐	For null hypothesis testing, the test statistic (e.g. *F*, *t*, *r*) with confidence intervals, effect sizes, degrees of freedom and *P* value noted<br>*Give P values as exact values whenever suitable.*
☒	☐	For Bayesian analysis, information on the choice of priors and Markov chain Monte Carlo settings
☒	☐	For hierarchical and complex designs, identification of the appropriate level for tests and full reporting of outcomes
☒	☐	Estimates of effect sizes (e.g. Cohen's *d*, Pearson's *r*), indicating how they were calculated

Our web collection on statistics for biologists contains articles on many of the points above.

Software and code

Policy information about availability of computer code

Data collection	Semi-structured interviews conducted via Microsoft Teams, transcribed via Microsoft Word
Data analysis	Transcripts analyzed via NVivo 12

For manuscripts utilizing custom algorithms or software that are central to the research but not yet described in published literature, software must be made available to editors and reviewers. We strongly encourage code deposition in a community repository (e.g. GitHub). See the Nature Portfolio guidelines for submitting code & software for further information.

Data

Policy information about availability of data

All manuscripts must include a data availability statement. This statement should provide the following information, where applicable:

- Accession codes, unique identifiers, or web links for publicly available datasets
- A description of any restrictions on data availability
- For clinical datasets or third party data, please ensure that the statement adheres to our policy

Broader access to the data reported in this analysis is restricted; data sharing was not part of the informed consent agreement.

Human research participants

Policy information about studies involving human research participants and Sex and Gender in Research.

Reporting on sex and gender	The study analyzed experiences and needs of patients living with prostate cancer, which is only applicable for biologically male participants. Gender information of participants was not collected. Therefore, sex/gender-based analysis was not performed.
Population characteristics	Population characteristics were collected and reported in the results section of the paper and a detailed table is presented as an appendix file.
Recruitment	Participants were recruited from three clinical participating sites, including participants from both urban and remote settings. A convenience sample was taken for both groups, and the relevant bias was addressed by thematic saturation strategy to ensure sufficient understanding and exploration of experiences and needs.
Ethics oversight	Ethics approval was obtained through University Health Network, which is a public research and teaching hospital network in Toronto, Ontario, Canada.

Note that full information on the approval of the study protocol must also be provided in the manuscript.

Field-specific reporting

Please select the one below that is the best fit for your research. If you are not sure, read the appropriate sections before making your selection.

☐ Life sciences ☒ Behavioural & social sciences ☐ Ecological, evolutionary & environmental sciences

For a reference copy of the document with all sections, see nature.com/documents/nr-reporting-summary-flat.pdf

Behavioural & social sciences study design

All studies must disclose on these points even when the disclosure is negative.

Study description	The study is a qualitative descriptive study.
Research sample	A convenience sample of 10 healthcare providers and 10 prostate cancer survivors (20 participants total) living in Ontario, Canada was recruited.
Sampling strategy	The sample was recruited from both urban and rural settings. The representativeness of the sample is not claimed as the purpose of this qualitative study was not to generalize the findings to broader populations. Instead, the study was designed to explore the participant experiences in the relevant contexts with transferability encouraged through thematic saturation during recruitment, thick description, appropriate analysis under the guidance of relevant conceptual frameworks, and transparency in reporting.
Data collection	Participant recruitment, informed consent processes, and data collection were conducted virtually. Data was collected via semi-structured interviews, each lasting about 60 minutes. Interviews were audio-recorded, then transcribed verbatim via transcription software (Microsoft Word, Redmond, Washington). Resulting transcripts were checked for accuracy by a member of the research team.
Timing	Data collection was conducted between May to June 2021.
Data exclusions	All data collected were included in the analysis and reporting.
Non-participation	All participants voluntarily consented to take part in the study and the study only involved one study visit, therefore non-participation data is not applicable.
Randomization	There was no randomization design in the study as it is not applicable for study purposes.

Reporting for specific materials, systems and methods

We require information from authors about some types of materials, experimental systems and methods used in many studies. Here, indicate whether each material, system or method listed is relevant to your study. If you are not sure if a list item applies to your research, read the appropriate section before selecting a response.

Materials & experimental systems

n/a	Involved in the study
☒	☐ Antibodies
☒	☐ Eukaryotic cell lines
☒	☐ Palaeontology and archaeology
☒	☐ Animals and other organisms
☒	☐ Clinical data
☒	☐ Dual use research of concern

Methods

n/a	Involved in the study
☒	☐ ChIP-seq
☒	☐ Flow cytometry
☒	☐ MRI-based neuroimaging
